# Voice quality changes after functional endoscopic sinus surgery in patients with nasal polyps

**DOI:** 10.1038/s41598-022-25841-8

**Published:** 2022-12-08

**Authors:** Jakkree Naruekon, Pornthep Kasemsiri, Sanguansak Thanaviratananich, Benjamas Prathanee, Cattleya Thongrong, Wisoot Reechaipichitkul

**Affiliations:** 1 Department of Otorhinolaryngology, Faculty of Medicine, Srinagarind Hospital, Khon Kaen University, Khon Kaen, Thailand; 2 Srinagarind Minimally Invasive Surgery Center of Excellence, Khon Kaen, Thailand; 3 Khon Kaen Head and Neck Oncology Research, Khon Kaen, Thailand; 4 Department of Anesthesiolology, Faculty of Medicine, Srinagarind Hospital, Khon Kaen University, Khon Kaen, Thailand; 5 Skull Base Surgery Unit, Department of Otorhinolaryngology, Faculty of Medicine, Srinagarind Hospital, Khon Kaen University, Khon Kaen, Thailand

**Keywords:** Diseases, Health care, Medical research

## Abstract

Nasal polyps are associated with hyponasality. The effect of functional endoscopic sinus surgery (FESS) on voice quality has not been adequately investigated; therefore, this study developed objective and subjective measurements to compare nasal polyp patients pre- and postsurgery. An observational prospective study was conducted at Srinagarind Hospital, Khon Kaen University, Thailand. Bilateral nasal polyposis patients who underwent FESS between August 1, 2015 and August 1, 2017, were recruited. All participants were assessed for nasal polyp grade, nasometry, acoustic parameters, acoustic perception, and patient satisfaction before surgery and at the 1-, 3- and 6-month follow-ups. Forty-six patients, 29 males and 17 females (mean age 48.2 years ± 16.2 years), were enrolled. Mean nasometry scores were significantly improved at 1, 3 and 6 months after surgery (p < 0.05), whereas the acoustic parameters were not significantly different after surgery (p > 0.05). Overall acoustic perception, assessed with a set of words and sentences, showed significant improvement in hyponasality voice after surgery (p < 0.05), whereas GIRBAS showed no significant change after surgery in each parameter of perception (p > 0.05). Patient satisfaction with voice changes after surgery was high, with significantly increased mean scores between the 1- and 6-month follow-ups (p < 0.05). The results showed that FESS for nasal polyposis patients improved voice quality and patients’ voice satisfaction ratings.

**Trial registration**: This trial was registered at the Thai Clinical Trial Registry (TCTR20210324004).

## Introduction

Quality of voice has an important role in social communication. Voice is produced by air from the lungs passing through the glottis that affects the vibration of the true vocal fold. Subsequently, the voice is modified by the supraglottis, pharynx, sinonasal tract, and oral cavity. These normal upper airway passages affect the resonance and articulation of voice; therefore, obstructive lesions or surgical procedures involving any of these upper airway structures may affect voice quality.

A nasal polyp is inflammation of the sinonasal mucosa that produces swollen mucosa that occludes the nasal passage and disturbs nasal resonance. Generally, functional endoscopic sinus surgery (FESS) has been introduced for the removal of nasal polyps and to widen the sinus ostium; therefore, nasal volumes are changed^1^, which affects nasal resonance and voice quality. Several studies showed significantly increased nasal resonance^2–5^ in patients who underwent FESS compared with prior surgery. However, there are conflicting data on voice quality in acoustic parameter analysis.

The acoustic parameters included the fundamental frequency (F0), jitter, shimmer, and NHR. The fundamental frequency (F0) is defined as the number of times a sound wave produced by the vocal folds repeats during a given time that referred to the number of cycles of opening or closing of the glottis^6^. Jitter is defined as the frequency variation from cycle to cycle, and shimmer is defined as the amplitude variation of sound wave^7^. The last parameter, NHR, is the parameter of the ratio between periodic and nonperiodic components comprising a segment of voice speech^8^. The first component arises from the vibration of the vocal fold, and the second follows from the glottal noise^6^. These acoustic parameter abnormalities seemed to represent the lesion at the glottic level; thus, FESS might not affect acoustic parameters.

Majidi et al.^5^ found no significantly increased shimmer and F0 frequency in the pre- and postsurgery groups. Regarding jitter and NHR, these parameters were not significantly changed after FESS. In contrast, Acar et al.^9^ showed that the postoperative changes in shimmer values between completely or near completely obstructing nasal cavity patients and other patients were statistically significant. They mentioned that the resulting nasal resonance changes could cause the development of adaptive mechanisms large enough to affect the voice in phonatory or resonatory systems. Thus, these acoustic parameters need to be reinvestigated. Moreover, subjective measurement of voice is also a very important test of voice functionality; however, there is a lack of data on this perception after endoscopic sinus surgery.

According to long-term voice quality after surgery, there was insufficient information; therefore, we designed to compare the voice quality of prior surgery with follow-up at three times until 6 months, which was different from previous studies^2–5,9^ that compared with a one-time follow-up in the postoperative period. Moreover, we evaluated the voice quality in multiple aspects, including objective tests (nasometry and reinvestigated acoustic parameters) and subjective tests (acoustic perception and patient satisfaction).

## Material and methods

This observational pre- and postsurgery prospective study was conducted between August 2015 and August 2017 on a consecutive series of patients presenting with bilateral nasal polyposis. Patients older than 18 years who presented with bilateral nasal polyposis with scheduled functional endoscopic sinus surgery (FESS) were regarded as eligible for inclusion. Exclusion criteria included patients with any of the following: previous history of cleft palate and/or submucosal cleft, had previously undergone FESS with tonsillectomy or uvulopalatopharyngoplasty, or had laryngeal disease or previous history of laryngeal surgery.

A sample size of 46 patients, with allowance for 10% loss to follow-up, was calculated as appropriate (95% confidence interval, 2% error). A correlation coefficient for repeated measures of 0.5, a mean difference nasal resonance of 3, and a standard deviation of nasal resonance of 6.9 was estimated from the study of Hong et al.^2^.

The day before surgery, all patients were examined by nasal endoscopy and laryngoscopic examination by one rhinologist to grade nasal polyposis (no polyp; grade I: polyp in middle meatus; grade II: polyp extended through middle meatus; grade III: polyp completely obstructed nasal cavity). Patients’ Lund-Mackay scores were obtained by preoperative computerized tomography (CT) scans of the paranasal sinus. Measures to compare quality of voice on nasometry, acoustic parameter analysis, and acoustic perception were obtained from all patients.

### Surgical procedure

The FESS was performed under the standard protocol for general anaesthesia. The nasal polyp was removed with a microdebrider, and widening of the sinus ostium and ethmoidectomy were performed individually based on preoperative CT scan findings. After surgery, all patients were prescribed standard medications, including two puffs of nasal steroid twice a day, antihistamine, and corticosteroid nasal irrigation. All patients visited the clinic for nasal cleaning 1 week after surgery. To evaluate postsurgery nasal endoscopy findings and laryngoscopic examination, all patients were scheduled for clinical follow-up at 1, 3 and 6 months**.**

### Voice analysis

Nasal resonance was assessed by a Nasometer II: Model 6450 (KayPENTAX™, Lincoln Park, NJ, USA) with a headset, sound filter, and computer analyst. The patient wore the headset with a baffle plate that fits the upper lip and cheek to separate the nasal passage and oral cavity. The two microphones were set on the superior and inferior surfaces of the baffle plate to detect acoustic energy from the nasal and oral cavities separately during speech. The three passage stimuli included: the nasal sentence (Winter) that assessed the full range of nasal consonants, the oral sentence (Laying Hen) designed to be devoid of nasal consonants, and the last sentence (My house) that assessed a mixture of oral and nasal consonants. The original three passages were in English; however, a modified Thai version with a Thai reference value^10,11^ was used in this study.

Acoustic parameters were assessed with the computerized speech lab (CSL): Model 4500 (KayPENTAX™, Lincoln Park, NJ, USA). The distance between the patient and a microphone was fixed at 10 cm in a quiet room. Vowel sounds were vocalized and sustained for at least five seconds with a comfortable pitch and loudness. The three seconds of the mid-vowel sound segment was selected for assessment of the four acoustic parameters, including jitter, noise to harmonic ratio (NHR), F0, and shimmer.

**Figure 1:**
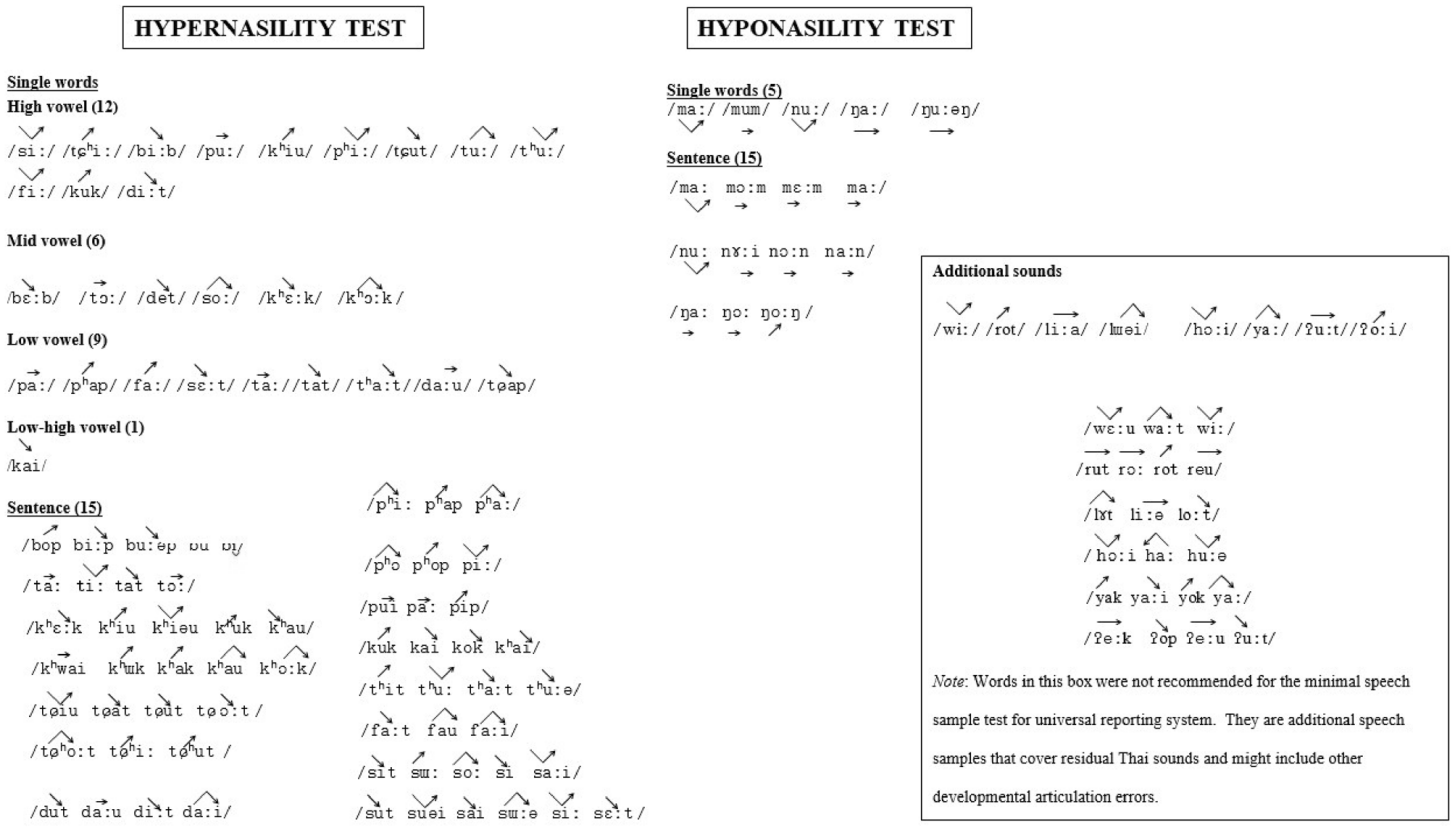
Set of words and sentences used for the acoustic perception test.

For subjective measurement, each patient read a standard set of words and sentences (Fig. 1) to assess hypo/hypernasality. The GIRBAS scale (grading, instability, roughness, breathiness, asthenia and strain) was also used to assess the voice perception test from counted numbers after the patient took a deep breath. The GIRBAS was accepted as a reliable perceptual scale^5,9^ with a range of 0–3 (0 = normal; 1 = slightly disturbance; 2 = moderate disturbance; 3 = severe disturbance)^12^. These tests were carried out by an experienced speech therapist who was masked to the nasometer and CSL results, subjective measurement outcomes at each visit, and the patient’s nasal polyposis details. Each patient also self-rated their postoperative voice quality satisfaction on a visual analogue scale (VAS), 0 (very dissatisfied) to 10 (extreme satisfaction). All tests were performed on each patient 1 day before surgery and at 1, 3 and 6 months after surgery.

### Data analyses

Descriptive data are presented as percentages and means ± SDs. ANOVA with Dunnett’s correction was used to compare the mean score of acoustic parameters and nasometry analysis pre- and postsurgery. Ordinal logistic regression was used to compare proportional nasality events pre- and postsurgery. A value of p < 0.05 was considered statistically significant. All data were analysed using STATA (v 10.1: Stata Corp. 2015, Texas, USA).

### Ethical review

The study was reviewed and approved by the Human Ethics Research Committee of Khon Kaen University (HE581296). The study was also registered with the Thai Clinical Trial Registry (TCTR20210324004). Informed consent was obtained from all patients following the Declaration of Helsinki.

### Ethics approval and consent to participate

This study was approved by Khon Kaen University Ethics Committee for Human Research (HE581296). All participants provide written consent following the Declaration of Helsinki before enrolment.

## Results

Forty-six nasal bilateral polyposis patients participated (29 males and 17 females, mean age 48.2 ± 16.2 years). According to nasal endoscopic findings and preoperative CT scans, 76% presented with nasal polyp grades II–III on the right side, whereas 80.5% presented with nasal polyp grades II–III on the left side. The mean Lund Mackay score on preoperative CT scans was 8.1 (Table 1).

**Table 1:** Demographic data.

Characteristic		Number	Percentage
**Gender**			
Male		29	63.0
Female		17	37.0
Age (mean ± SD)		48.2 ± 16.2	
**Underlying disease**			
None		21	45.7
Diabetes mellitus		1	2.2
Hypertension		7	15.2
Allergy		6	13.0
Other		15	32.6
**Diagnosis**			
Right nasal polyp	Grade I	11	23.9
	Grade II	12	26.1
	Grade III	23	50.0
Left nasal polyp	Grade I	9	19.6
	Grade II	17	37.0
	Grade III	20	43.5
**Lund Mackay score (mean ± SD)**			
Right		8.2 ± 3.0	
Left		8.1 ± 3.0	

All patients received bilateral full-house FESS. After surgery, 4, 6 and 9 patients were lost to follow-up at 1, 3 and 6 months, respectively. Regarding postoperative nasal endoscopic findings, most patients presented with an enlarged maxillary ostium and sphenoid sinus of more than 4 mm, whereas the frontal recess in half of our patients could not be identified due to mucopolypoid changes and polyps obscuring the recess. Most patients had no residual or recurrent nasal polyps in the ethmoid, maxillary or sphenoid sinus at the 6-month follow-up (Table 2). Furthermore, our patients were observed to have no glottis problems pre- and postsurgery.

**Table 2:** Nasal endoscopic findings after surgery.

Characteristics	Nasal endoscopic assessment after surgery					
	1 month (N = 42)		3 months (N = 40)		6 months (N = 37)	
	Right (N)	Left (N)	Right (N)	Left (N)	Right (N)	Left (N)
**Maxillary sinus**						
Ostium						
Cannot identify	4	4	3	3	7	7
< 4 mm	4	9	5	4	2	3
≥ 4 mm	34	29	32	33	28	27
Residual/recurrent nasal polyp						
No polyp	32	30	29	30	28	26
Mucopolypoid change	9	11	10	9	7	8
Numerous	1	1	1	1	2	3
**Ethmoid sinus**						
Residual/recurrent nasal polyp						
No polyp	28	29	29	27	20	23
Grade I	14	12	6	9	14	11
Grade II	0	1	5	2	2	1
Grade III	0	0	0	2	1	2
**Frontal sinus**						
Frontal recess						
Cannot identify	19	17	18	17	21	20
< 4 mm	7	6	4	2	3	6
≥ 4 mm	16	19	18	21	13	11
Residual/recurrent nasal polyp						
No polyp	23	18	23	20	18	15
Mucopolypoid change	18	23	13	15	15	17
Numerous	1	1	4	5	4	5
**Sphenoid sinus**						
Ostium						
Cannot identify	12	14	11	11	11	7
< 4 mm	4	1	2	0	6	7
≥ 4 mm	26	27	27	29	20	23
Residual/recurrent nasal polyp						
No polyp	27	29	29	26	25	26
Mucopolypoid change	14	12	7	9	9	9
Numerous	1	1	4	5	3	2

**Figure 2:**
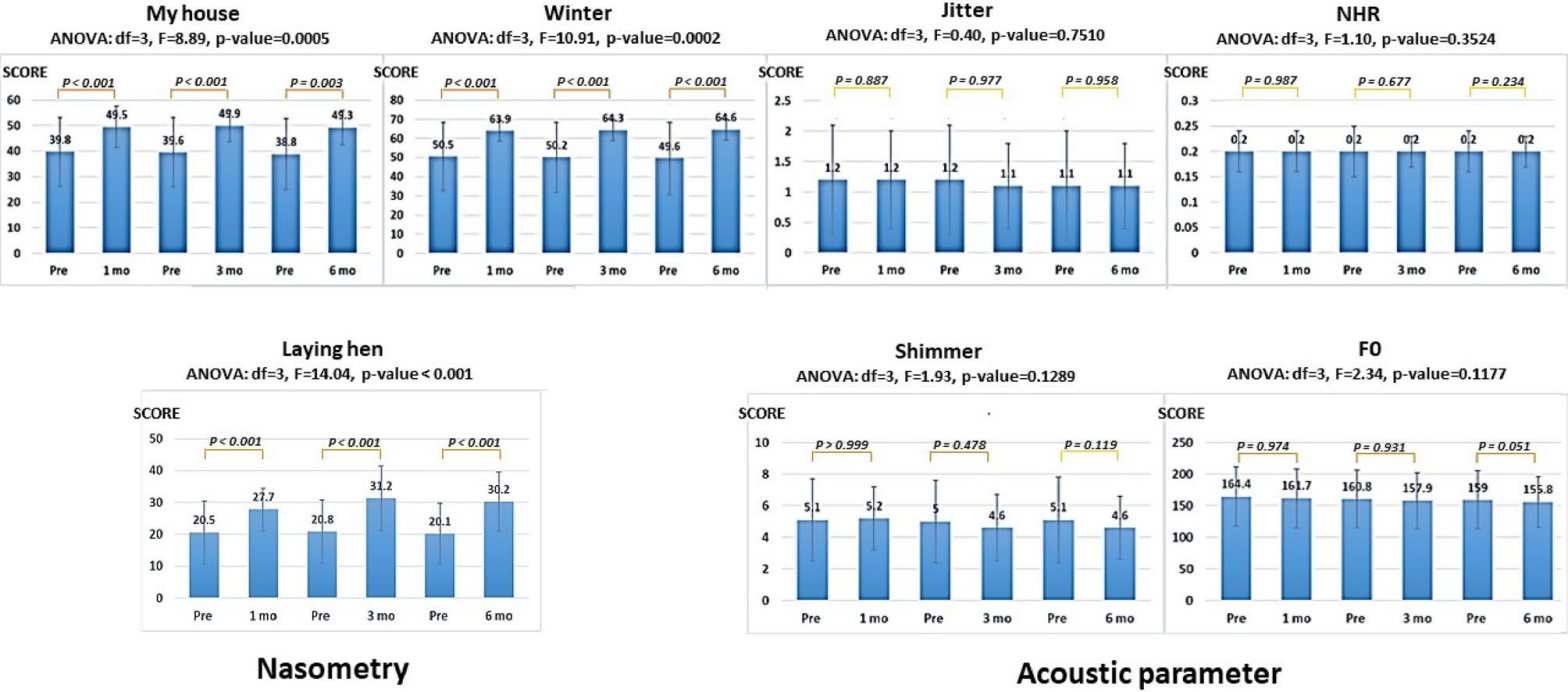
Objective measurement of voice included nasometry and acoustic parameters. All parameters of nasometry showed that the mean score between pre- and postsurgery was significantly different at 1, 3 and 6 months (p < 0.05); in contrast, all acoustic parameters showed no significant difference pre- and postsurgery at 1, 3 and 6 months (p > 0.05).

Nasometry showed a significant increase in mean scores for all three passages after surgery (p < 0.05), whereas the acoustic parameters showed no significant difference pre- and postoperation at 1, 3 and 6 months (p > 0.05) (Fig. 2).

**Figure 3:**
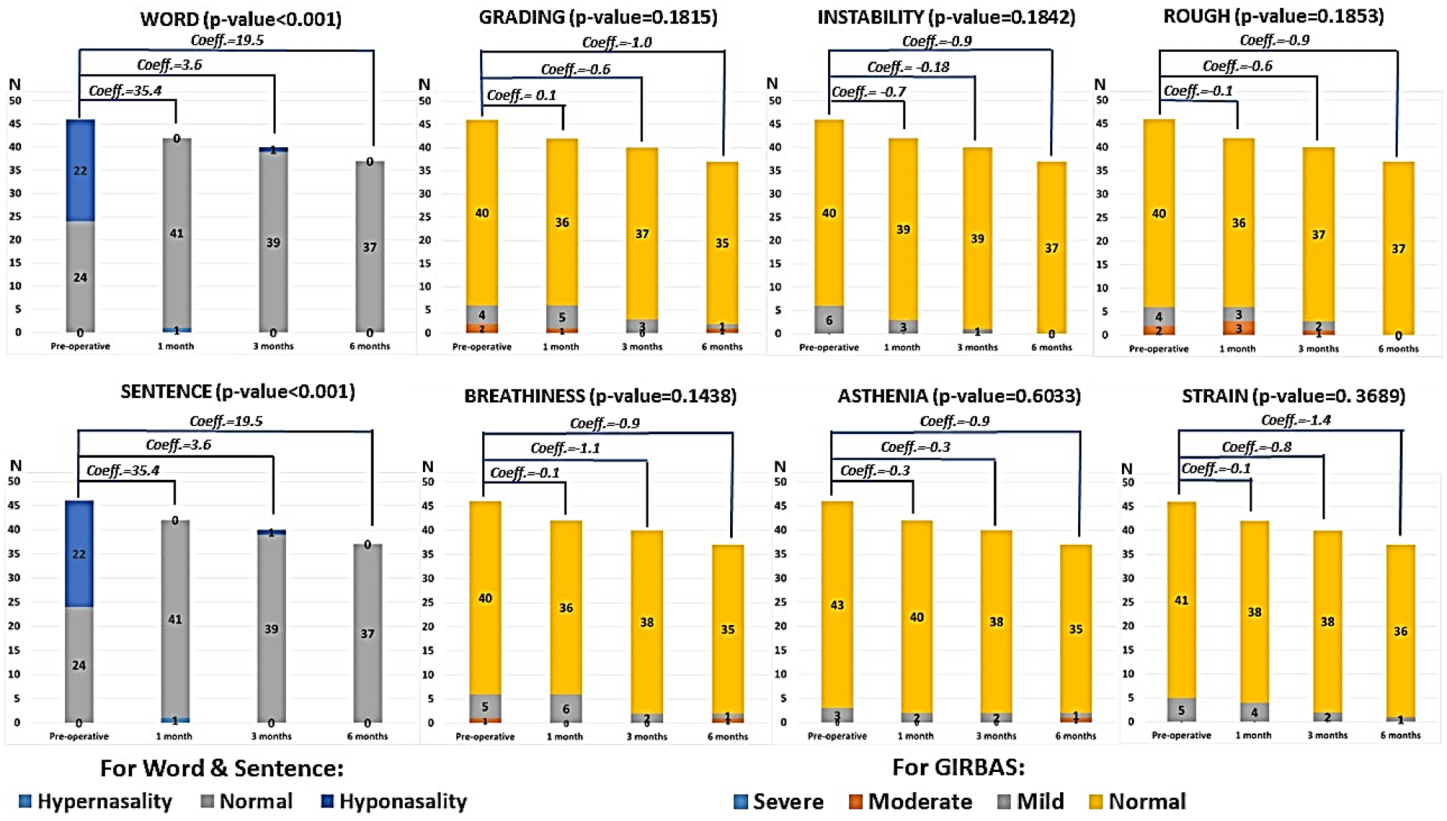
Subjective measurement of voice analysis showed that the number of patients who had the set word and sentence were improved from hypo to normal nasality at 1, 3 and 6 months after surgery (p < 0.001). The GIRBAS scale after surgery did not significantly change from prior surgery (p > 0.05).

On subjective measures, the set words and sentences analysis showed that half of the patients were hyponasal presurgery but made a statistically significant improvement to normal nasality after surgery (p < 0.001). The GIRBAS scale showed no significant acoustic perceptual difference between the pre- and postoperative periods (p > 0.05) (Fig. 3).

**Figure 4:**
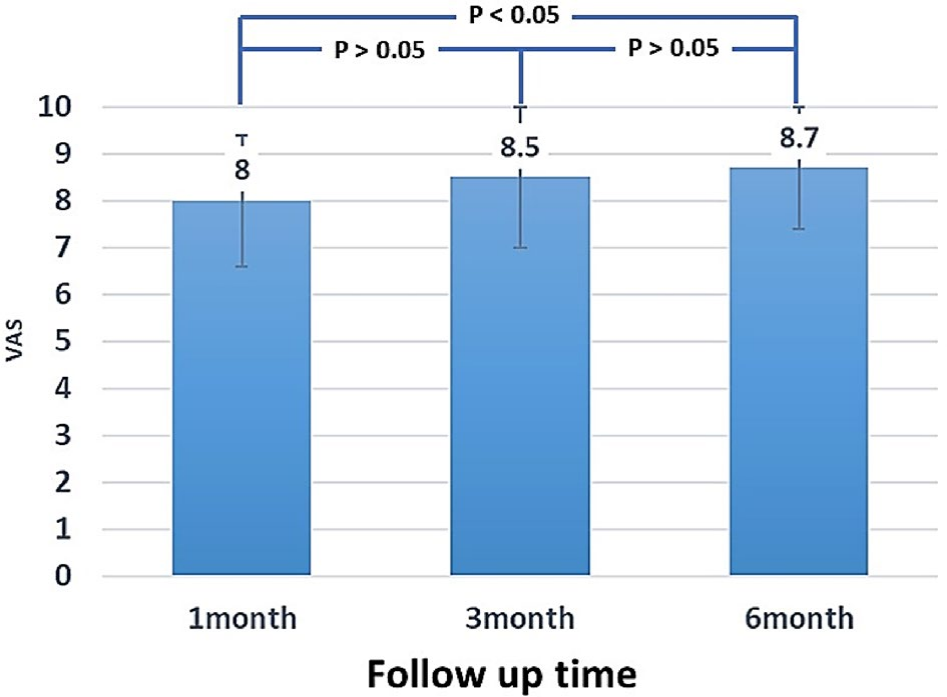
The visual analogue score of patients’ satisfaction with their voice change after surgery revealed a mean score of 8.0 ± 1.4 at 1 month, 8.5 ± 1.5 at 3 months, and 8.7 ± 1.3 at 6 months. A significantly different mean score between 1 and 6 months was observed (p < 0.05).

The postsurgery patient satisfaction VAS scores consistently increased with longer follow-up, with mean scores of 8.0, 8.5 and 8.7 at the 1-, 3-, and 6-month follow-ups, respectively (Fig. 4). Six months after surgery, VAS mean scores were significantly increased over the 1-month postsurgery follow-up (p < 0.05).

## Discussion

The quality of voice is one of the most important factors that affects communication in social life. The sinonasal complex contributes to the resonance of the voice; therefore, endoscopic removal of nasal polyposis can affect nasal resonance and lead to voice quality changes. In the present study, nasometry showed statistically significant increased mean scores for three reading passages, similar to several previous studies. Arslan et al.^3^ also found significantly increased nasal resonance scores for those three passages (P < 0.001). Similarly, Jiang et al.^4^ found statistically significant increased nasal resonance scores after surgery for chronic sinusitis patients with or without nasal polyps (p < 0.05). The effect of sinus surgery was a nasal volume change that led to nasal resonance changes. Jiang et al.^4^ found a significant correlation between increased nasal resonance scores and larger nasal volumes (p < 0.05). FESS removed the nasal polyp that caused nasal passage obstruction and widened the sinus ostium, which increased nasal volume, thus leading to increased nasal resonance scores.

From acoustic parameter analyses, our results suggested that FESS had no significant effect on changing acoustic parameters because the acoustic parameters mainly reflected glottic pathology. In our study, the patients were observed to have no problems with the glottis before and after surgery. It might be concluded that FESS did not significantly affect acoustic parameters. These results are similar to Majidi et al.^5^, who found no significant difference between pre- and postoperation.

Acoustic perception is the other important factor that impacts speech communication in social life. The GIRBAS scores in the present study showed no significant changes from baseline, similar to Kim et al.^13^. They found that the GIRBAS scales in all patients changed by less than two scales postoperatively. However, the sets of words and sentences in our study revealed a statistically significant improvement to normal nasality (p < 0.05) at 1, 3, and 6 months after surgery. Furthermore, there was one patient who presented with hypernasality in the first month after FESS and returned to normal 2 months later. In this case, very large bilateral nasal polyps extending to the choana and nasopharynx were observed. The polyps might compress the velopharyngeal valve for a long time; therefore, hypernasality occurred after removing the polyps in the first month. However, the velopharyngeal valve could be adaptively compensated to return to normal activity at the third month after surgery.

Regarding patients’ voice quality satisfaction ratings, our study showed that they consistently increased at 1, 3, and 6 months after FESS, with a significant difference between the 1- and 6-month follow-ups (p < 0.05). These findings correlate well with Arslan et al.^3^, who measured voice perception with the voice handicap index. They reported a significant improvement in the mean VHI-10 score (p < 0.001). Therefore, our study further affirms that endoscopic sinus removal of nasal polyps allows for improved acoustic perception after surgery. Unfortunately, our nine patients (19.5%) were lost to follow-up at the sixth month, which limited the evaluation of the quality of voice in the long-term postoperative periods. Further study should be developed to address this point. Another possible limitation of our study is the use of subjective acoustic perceptual measurements, including the GIRBAS scale and the set of words and sentences, by using one experienced speech therapist due to the lack of a speech therapist in our institution. However, we attempted to control for bias with a masked speech therapist from subjective and objective test results at each visit and patient clinical and surgery details.

## Conclusion

Endoscopic surgical treatment in nasal polyposis patients allowed significantly increased nasometry with better acoustic perception, which improved hyponasality to normal nasality within the first month until the sixth month follow-up. These results were correlated with better patient satisfaction after surgery. Furthermore, the insignificantly changing acoustic parameters showed that surgery did not significantly disturb the voice at the glottic level. Therefore, our results should be encouraging for the surgical treatment of nasal polyposis in professional voice users.

## Data Availability

The datasets used and/or analyzed during the current study are available from the corresponding author on reasonable request.

## Abbreviations

FESS: Functional endoscopic sinus surgery
CT: Computed tomography
Coeff.: Coefficient
df: Degree of freedom
